# Glucagon-like Peptide-2 Acts Partially Through Central GLP-2R and MC4R in Mobilizing Stored Lipids from the Intestine

**DOI:** 10.3390/nu17091416

**Published:** 2025-04-23

**Authors:** Kundanika Mukherjee, Muhammad Saad Abdullah Khan, John G. Howland, Changting Xiao

**Affiliations:** Department of Anatomy, Physiology and Pharmacology, College of Medicine, University of Saskatchewan, 107 Wiggins Road, Saskatoon, SK S7N 5E5, Canada; kum581@mail.usask.ca (K.M.); knf875@mail.usask.ca (M.S.A.K.); john.howland@usask.ca (J.G.H.)

**Keywords:** glucagon-like peptide-2, GLP-2 receptor, melanocortin 4 receptor, gut-brain neural pathway, triglycerides, chylomicrons, transcriptomics

## Abstract

**Background:** Glucagon-like peptide-2 (GLP-2) is a gut hormone secreted in response to nutrient intake and regulates lipid metabolism in the gut. The present study aims to elucidate the underlying mechanism of GLP-2 in stimulating gut lipid secretion in the fasted state by testing whether GLP-2 signals through the brain’s GLP-2 receptor and melanocortin 4 receptor (MC4R). **Methods:** Sprague-Dawley rats were implanted with a mesenteric lymph duct cannula for measuring gut lipid secretion and an intracerebroventricular cannula for infusion of a GLP-2R antagonist (GLP-2(11-33)), an MC4R antagonist (SHU9119), or saline as a control. The rat received a lipid infusion into the small intestine and a peritoneal injection of GLP-2 five hours later. **Results:** Brain administration of a GLP-2R antagonist or an MC4R antagonist attenuated the stimulatory effects of peripheral GLP-2 on lymph triglyceride output. These effects were associated with differential changes in the expression of key genes in jejunal endothelial cells, smooth muscle cells, and neuronal cells. **Conclusions:** These results support the involvement of central GLP-2R and MC4R in a neural pathway for GLP-2 to mobilize lipids stored in the gut during the post-absorptive state.

## 1. Introduction

Glucagon-like peptide-2 (GLP-2) is a gut hormone secreted in response to nutrient ingestion and regulates gut lipid handling. Recent studies demonstrate that its regulation of gut lipid handling invokes neural pathways; however, the underlying mechanisms, especially its signaling in the brain, remain poorly understood. GLP-2 regulates gut lipid handling differently during the postprandial state and the postabsorptive state. During the postprandial state, GLP-2 increases dietary fat absorption and chylomicron formation in enterocytes [1,2]. In the absence of lipid absorption during the fasted state, GLP-2 releases “pre-formed” chylomicrons stored in locations outside the enterocytes [3,4,5].

There is evidence of the involvement of neural pathways in GLP-2-mediated regulation of gut lipid handling. The GLP-2 receptor (GLP-2R) is expressed by enteric neurons [6], the vagus nerve [7], central neurons [8,9,10], and intestinal myofibroblasts [11], but not on enterocytes [6]. These receptor expression patterns support that GLP-2 may regulate gut lipid handling by enlisting neural components locally in the gut and centrally in the brain. Indeed, GLP-2 stimulation of postprandial lipid absorption is mediated by neuronal nitric oxide synthase [12]. The mechanism for GLP-2 in mobilizing stored lipids from the gut during the post-absorptive state is not well defined [13]. In a recent study, we showed that a CNS-dependent neural pathway is involved in this process [14]. Specifically, we performed subdiaphragmatic vagotomy to cause the loss of the gut-brain neural communication. This loss of communication attenuated the effects of peripheral GLP-2 on the stimulated release of lipids stored in the gut during the post-absorptive state. The nature of this pathway, such as its key components in the brain, is not known.

Several pieces of evidence support brain GLP-2R and melanocortin 4 receptor (MC4R) as potential key players in this neural pathway. GLP-2 regulates feeding behavior and GI function through central GLP-2R and the melanocortin system [15]. Specifically, hypothalamic GLP-2R and brainstem MC4R are downstream targets of the GLP-2/GLP-2R signaling pathway in regulating food intake and gastric emptying [15]. MC4R is expressed in multiple organs, including the brain, and central MC4R is well-known for the regulation of feeding behavior and adipose metabolism [16]. Particularly, hypothalamic GLP-2R activation activates the brainstem region where MC4R is expressed, and both central GLP-2R and MC4R regulate gastric motility [15,17]. Hence, we hypothesized that peripheral GLP-2 acts through brain GLP-2R and MC4R in mobilizing stored lipids from the gut. The objective of this study was to evaluate the effects of blocking brain GLP-2R or MC4R on lipid release from the gut by GLP-2 during the postprandial state in rats.

## 2. Materials and Methods

### 2.1. Peptides

[Gly^2^]GLP-2 (1–33) (a dipeptidyl peptidase IV-resistant GLP-2 analogue, referred to as GLP-2) and GLP-2(11-33) (a GLP-2R antagonist) were obtained from Pepceuticals Ltd. (Leicestershire, UK). SHU9119, an MC4R antagonist, was obtained from MedchemExpress (Monmouth Junction, NJ, USA).

### 2.2. Animals

Adult male Sprague-Dawley rats (200–350 g body weight, 8–10 weeks old) were obtained from Charles River Laboratories (Senneville, QC, Canada). Before surgery, rats in pairs were acclimatized for 2 weeks in polycarbonate cages in a room with controlled conditions (temperature, humidity, and automatic 12-h light/dark cycle). Rats were provided with ad libitum water and a standard laboratory diet (LabDiet, St. Louis, MO, USA; Prolab RMH 3000; calories provided by protein 26.1%, fat 14.4%, and carbohydrates 59.5%). All animal procedures were approved by the Animal Research Ethics Board of the University of Saskatchewan (Animal Use Protocol 20200080, 1 November 2021). All experimental methods and procedures involving animals adhered to the guidelines and regulations of the University Animal Care Committee of the University of Saskatchewan and complied with the ARRIVE guidelines.

### 2.3. Intracerebroventricular (icv) Cannulation

Two weeks following acclimatization, on the surgery day, the animals were weighed and injected with analgesics (buprenorphine, metacam and lidocaine). Stereotaxic surgery was performed for two separate groups of rats to implant a catheter (stainless steel guide cannula, 22 gauge; Plastics One, Roanoke, VA, USA) into the 3rd cerebral ventricle (anterior posterior = −2.70 mm, mediolateral = 1.59 mm, and dorsoventral = −8.15) or the 4th ventricle (anterior posterior = −11.80 mm, mediolateral = 0.00 mm, and dorsoventral = −6.65) using the coordinates of Paxinus and Watson Atlas [18,19,20].

### 2.4. Mesenteric Lymph Duct (MLD), Intraduodenal (id), and Intraperitoneal (ip) Cannulations

Following 1 week of recovery from icv surgery, all rats underwent MLD, id, and ip cannulations as previously described [14]. Post-surgery, the rats were transferred to Bollman restraint cages (in a ventilated and temperature-controlled (26 °C) incubator) and were infused intraduodenally with 5% glucose solution at a rate of 3 mL/h for 4 to 5 h. Overnight fasting was simulated by infusing saline without glucose. Saline infusion was maintained throughout the study to keep the rats hydrated and to maintain MLD catheter patency.

### 2.5. Study Design

**Figure 1:**
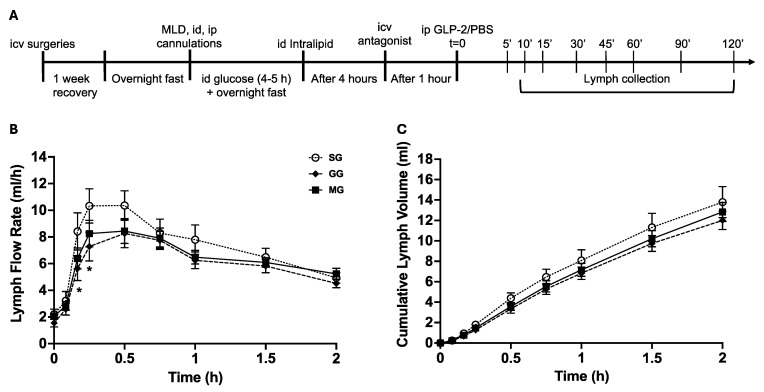
(**A**) Experimental timeline for determining the involvement of GLP-2R and MC4R in the GLP-2 effect of gut lipid mobilization. (**B**) Lymph flow rate (LFR) over time following GLP-2R or MC4R blockade. (**C**) Cumulative lymph volume over time following GLP-2R or MC4R blockade. Results are mean ± SEM: icv saline + ip GLP-2 (SG), n = 6; icv GLP-2(11-33) + ip GLP-2 (GG), n = 9; icv SHU9119 + ip GLP-2 (MG), n = 7. Two-way Repeated Measures (RMs) ANOVA followed by Tukey’s multiple comparison was used for statistical analysis. * *p* < 0.05, GG vs. SG.

Lipid secretion from the gut in response to GLP-2 during the fasted state was measured in rats pre-treated with brain administration of GLP-2R antagonist or MC4R antagonist. After an overnight fast, rats were infused with a lipid bolus (Intralipid 20%; 1.5 mL; Sigma-Aldrich, St. Louis, MO, USA) through the id catheter. Four hours later, rats randomly received icv infusion of the following compounds, with doses based on previously published literature: GLP-2R antagonist GLP-2(11-33) (15 μg, 2 μL, n = 9) [21], the MC4R antagonist SHU9119 (15 μg, 2 μL, n = 7) [19,22,23], or saline as control (2 μL, n = 6). Infusion was administered over 2 min through a Hamilton syringe using a syringe pump (Harvard Apparatus, Holliston, MA, USA). GLP-2(11-33) is a potent GLP-2R antagonist, with strong binding activity and without agonistic activity to rat GLP-2R, making it ideal for studies in rats [21]. Pre-treating mice with SHU9119 for an hour blocks central MC4R [24]. The timing of icv GLP-2(11-33) was chosen to be consistent with SHU9119. Five hours after the fat load (t = 0), i.e., during the fasted state, all rats received ip GLP-2 (75 μg in 1 mL PBS) to assess the effects of GLP-2 on releasing intestinal lipids (Figure 1A). The same dose of GLP-2 administered at the same timepoint elicited robust post-prandial lipid release from the intestine in the same animal model [3]. Lymph samples were collected at a series of time points following GLP-2 (5, 10, 15, 30, 45, 60, 90, and 120 min), corresponding to the time when GLP-2 elicits robust effects on releasing lipids from the intestine [3]. At the end of the experiment, rats were euthanized by sodium pentobarbital overdose. Jejunal tissues (the mid-segment of the small intestine) were immediately collected and flash frozen.

### 2.6. Lymph Flow Rate, Triglyceride and ApoB48 Assays, and Calculations of Gut Lipid Output

Recorded lymph volume (ml) was used to calculate lymph flow rate (LFR, mL/h), which is expressed as the lymph fluid volume per hour for each time point. Post-treatment gradual increase of lymph volume over time for 2 h was calculated to determine cumulative lymph volume (mL). Triglyceride (TG) output (mg/h) and cumulative TG amount were calculated as previously described [14]. Lymph apolipoprotein B48 (ApoB48) concentration (μg/mL) was determined using a rat ApoB48 ELISA kit (CUSABIO, Houston, TX, USA). ApoB48 amount (μg) and ApoB48 output (μg/h) were calculated using our previously established protocol [14].

### 2.7. Spatial Profiling of Jejunal mRNA

Fresh frozen jejunal tissues were sectioned in Tissue-Tek^®^ O.C.T Compound (Sakura, Torrance, CA, US) using a Leica microtome at 6 um thickness. The sectioned samples were mounted on Fisherbrand Superfrost Plus slides. The NanoString GeoMX™ Digital Spatial Profiling platform (NanoString Technologies, Seattle, WA, USA) was used for spatial transcriptomics analysis. Briefly, slide-mounted tissue sections were stained with different morphology markers, including NeuN for neuronal cells, CD31 for endothelial cells, SMA for muscle cells, and a nuclear stain (DNA). Samples were sequenced on a NovaSeq 6000, and sequencing data were processed using the GeoMX DSP software. To determine subsequent gene expression, areas of illumination (AOIs) were selected with the help of the morphology markers by visualizing the images of the stained slides in GeoMX^®^ Digital Spatial Profiler. Thirty AOIs segmented by NeuN, CD31, and SMA were selected for gene expression analysis. Within the selected AOIs, approximately 12,000 genes were detected, and they were assessed using the NanoString Mouse Whole Transcriptome Atlas.

### 2.8. Statistical Analysis

Data were presented as mean ± SEM. GraphPad Software (Version 9) was used for data analysis and representation. Time-course curves were analyzed using a Two-way Repeated Measures ANOVA followed by post-hoc Tukey’s multiple comparisons test.

For spatial transcriptomics, sequencing quality was inspected for sufficient saturation. Sequencing Saturation sets the minimum percentage of sequencing saturation allowed, calculated as 1% unique reads of a sample. 100% sequencing saturation indicates a representative sample, while 0% sequencing saturation indicates that all reads were unique. Below 50%, the counts are less reliable. To account for systematic variation between AOI’s each count was normalized by the 75th percentile (Q3) of expression for each AOI. To ensure normalization and quality control procedures were accurate, each AOI’s Q3 value was compared to its limit of quantification. Fold change and significance (in *p* < 0.05) are calculated based on log2 transformed Q3 normalized counts. Heat maps were generated using ComplexHeatMap (version 2.13.1) using the top 40 genes. Gene set enrichment analysis (GSEA) was implemented using the *fgsea* package in R. GSEA was leveraged to calculate normalized enrichment scores, identifying pathways significantly differentially expressed up or down in the context of three different sample sets.

## 3. Results

### 3.1. LFR and Cumulative Lymph Volume

The mesenteric lymph duct cannulated rat model was used to ensure direct collection of lymph from the gut. LFR reflects how fast lymph fluid is secreted from the intestine. As previously shown [14], LFR increased from baseline following ip GLP-2 and peaked at approximately 15–30 min. In rats receiving icv GLP-2R antagonist (GG), LFR also increased from baseline but was lower compared to rats treated with icv saline (SG) at 10 and 15 min after treatment (*p* < 0.05 GG vs. SG, Figure 1B). LFR in rats treated with icv MC4R antagonist (MG) also increased from baseline following treatment, but the increase was less pronounced than in SG, and the difference from SG was not statistically significant.

Cumulative lymph volume, i.e., the total lymph fluid secreted over time, increased from baseline following ip GLP-2 in all treatments. In GG rats, cumulative lymph volumes were similar to those in saline-treated rats. The MG group followed the same trend as the GG group and showed no statistically significant difference compared to the SG or GG groups (Figure 1C).

### 3.2. Lymph TG Output and Cumulative TG Amount

**Figure 2:**
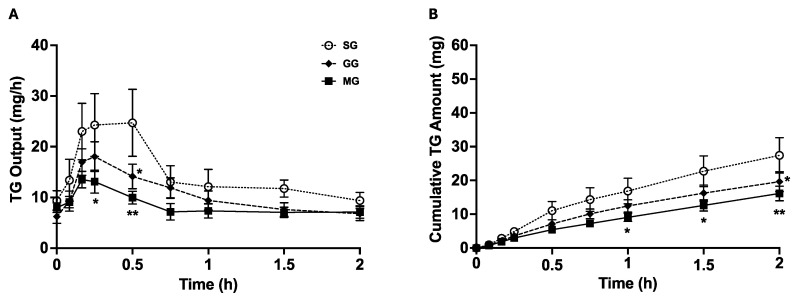
GLP-2 increases TG output and cumulative TG amount over time, acting through central GLP-2R and MC4R. (**A**) TG output over time following GLP-2R or MC4R blockade. (**B**) Cumulative TG amount over time following GLP-2R or MC4R blockade. Results are mean ± SEM: icv saline + ip GLP-2 (SG), n = 6; icv GLP-2(11-33) + ip GLP-2 (GG), n = 9; icv SHU9119 + ip GLP-2 (MG), n = 7. Two-way Repeated Measures (RMs) ANOVA followed by Tukey’s multiple comparison was used for statistical analysis. * *p* < 0.05, GG vs. SG or MG vs. SG; ** *p* < 0.001, MG vs. SG.

TG is the most abundant neutral lipid in chylomicrons. TG concentration in the lymph was first measured to assess chylomicron secretion. Lymph TG concentrations were not significantly different among the three groups (Supplement Figure S1). TG output increased from baseline following ip GLP-2 and peaked at approximately 15–30 min (Figure 2A). The GG group had significantly lower TG output at 30 min compared to the SG group (*p* < 0.05), with no difference at other time points. TG output was significantly lower in the MG group compared to the SG group within 15 min and lasted for approximately 30 min (*p* <0.001). In the MG group, TG output tended to be lower than the GG group but was not significantly different.

The amount of TG released over time was calculated to determine the cumulative TG amount (Figure 2B). Cumulative TG amount increased from baseline following ip GLP-2 in all treatments. In GG rats, the cumulative TG amount was lower compared to SG rats at 2 h following ip GLP-2 (*p* < 0.05, GG vs. SG). The cumulative TG amount in the MG group was significantly lower at 1 h (*p* < 0.05, MG vs. SG) and lasted till the end of the experiment (120 min, *p* < 0.001, MG vs. SG) compared to the SG group. The MG group followed the same trend as the GG group and had no statistical significance compared to the GG group.

### 3.3. Lymph ApoB48 Output and Cumulative ApoB48 Amount

**Figure 3:**
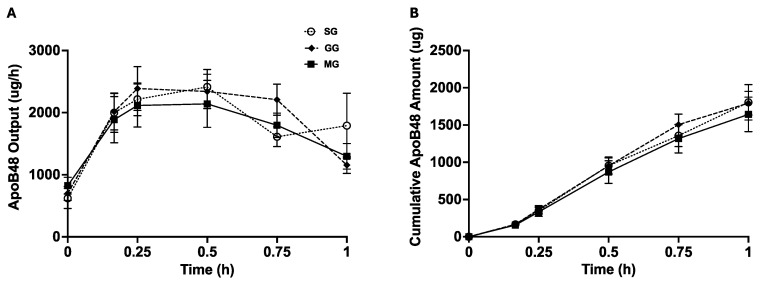
Involvement of central GLP-2R and MC4R in GLP-2-mediated mobilization of stored chylomicron particles. (**A**) ApoB48 output over time following GLP-2R or MC4R blockade. (**B**) Cumulative ApoB48 amount over time following GLP-2R or MC4R blockade. Results are mean ± SEM: icv saline + ip GLP-2 (SG), n = 6; icv GLP-2(11-33) + ip GLP-2 (GG), n = 9; icv SHU9119 + ip GLP-2 (MG), n = 7. Two-way Repeated Measures ANOVA followed by Tukey’s multiple comparison was used for statistical analysis.

ApoB48 is a structural protein for chylomicrons, and each chylomicron particle has one ApoB48. Quantification of lymph ApoB48 output and total ApoB48 amount therefore indicates the secretion rate of chylomicron particles and total number of chylomicron particles secreted over time. ApoB48 output and cumulative ApoB48 amount were not significantly different among the three groups (Figure 3). Together with lower TG outputs in GG and MG vs. SG, this suggests that icv GLP-2R and MC4R antagonism led to the secretion of smaller chylomicron particles without affecting particle numbers.

### 3.4. Spatial Transcriptomics Analysis

**Figure 4:**
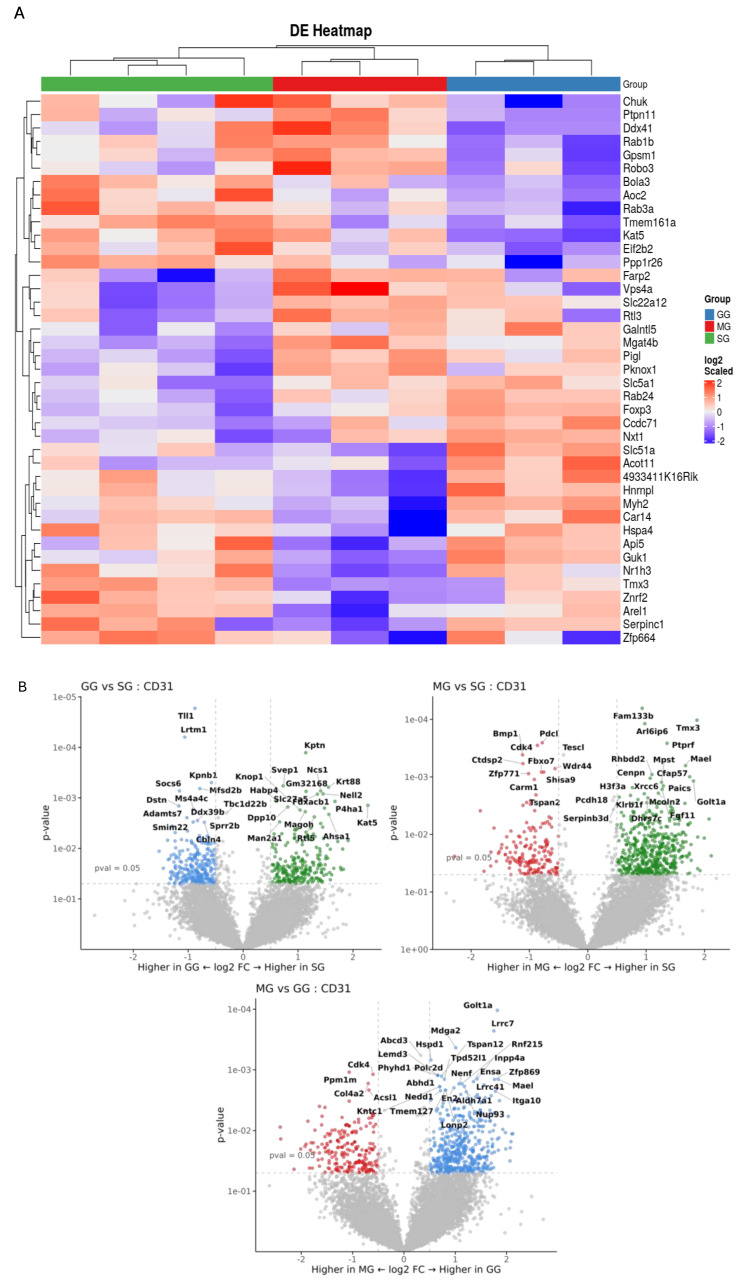
(**A**) Expression heatmap of differentially expressed genes in CD31 cells. In the heatmap, red denotes upregulated genes and blue downregulated genes. Each row represents relative gene expression. Each column represents data received from samples in between three treatments (SG, GG, MG). (**B**) Volcano plot representation of the differentially expressed genes between two treatments at a time. log2 Fold Change (FC) is on the x-axis, and *p*-value is on the y-axis. Each dot represents a gene and has been colored according to the log2FC. Grey indicates the genes that are not significantly upregulated or downregulated. Green indicates the genes that are upregulated in SG. Red indicates the genes that are upregulated in MG. Blue indicates the genes that are upregulated in GG.

**Figure 5:**
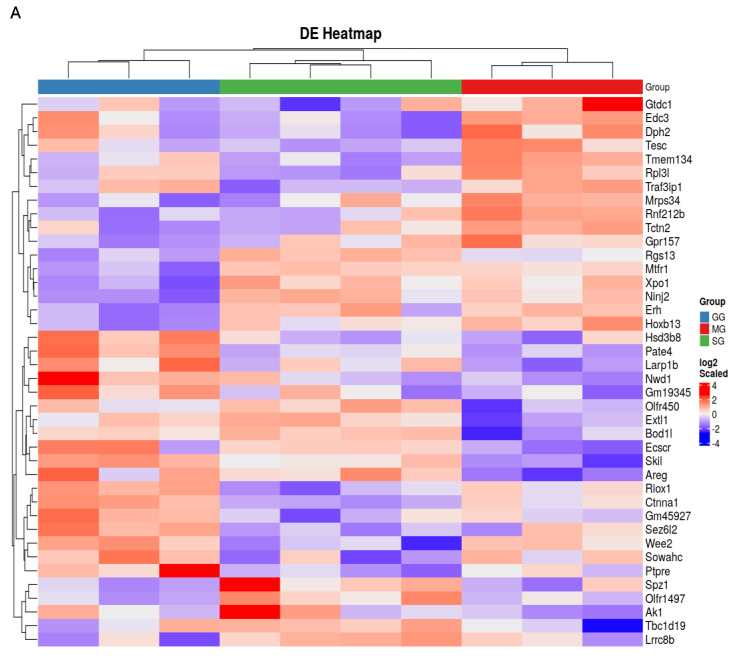

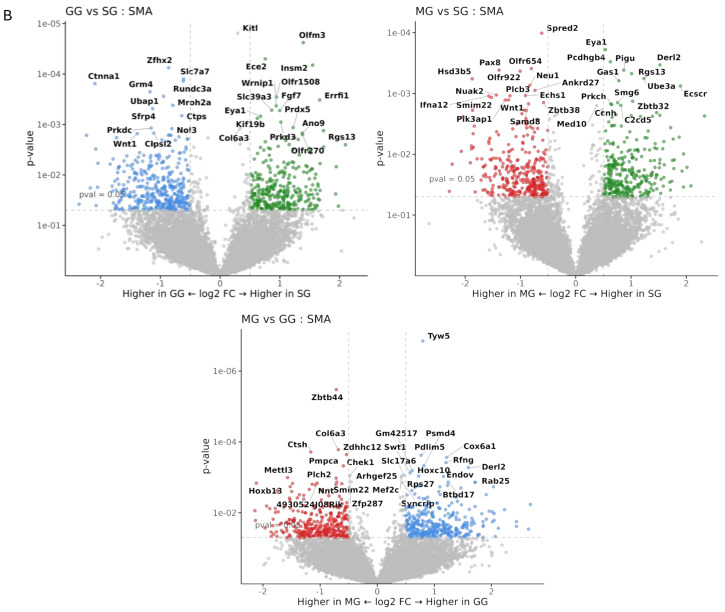
(**A**) Expression heatmap of differentially expressed genes in SMA cells. In the heatmap, red denotes upregulated genes and blue downregulated genes. Each row represents relative gene expression. Each column represents data received from samples in between three treatments (SG, GG, MG). (**B**) Volcano plot representation of the differentially expressed genes between two treatments at a time. log2 Fold Change (FC) is on the x-axis, and *p*-value is on the y-axis. Each dot represents a gene and has been colored according to the log2FC. Grey indicates the genes that are not significantly upregulated or downregulated. Green indicates the genes that are upregulated in SG. Red indicates the genes that are upregulated in MG. Blue indicates the genes that are upregulated in GG.

**Figure 6:**
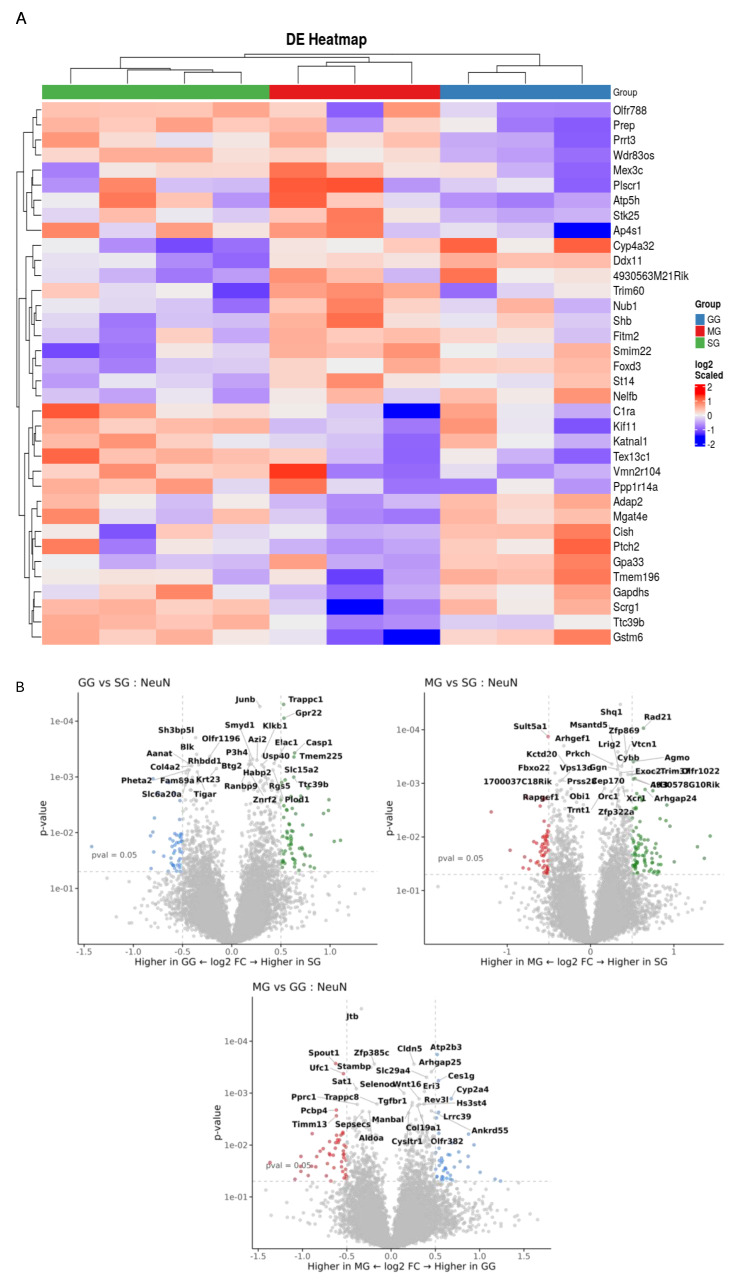
(**A**) Expression heatmap of differentially expressed genes in NeUN cells. In the heatmap, red denotes upregulated genes and blue downregulated genes. Each row represents relative gene expression. Each column represents data received from samples in between three treatments (SG, GG, MG). (**B**) Volcano plot representation of the differentially expressed genes between two treatments at a time. log2 Fold Change (FC) is on the x-axis, and *p*-value is on the y-axis. Each dot represents a gene and has been colored according to the log2FC. Grey indicates the genes that are not significantly upregulated or downregulated. Green indicates the genes that are upregulated in SG. Red indicates the genes that are upregulated in MG. Blue indicates the genes that are upregulated in GG.

Spatial transcriptomics analysis of the jejunal tissues were performed to gain mechanistic insights on the regulation by various treatments at the level of specific regions, cell types, and genes. Data quality and assay performance for the experiment were checked (Supplement Figure S2). Principal component analysis was performed to visualize the large-scale variability within each cell type between treatments (Supplement Figure S3). A total of 12,125 genes were detected across all 30 AOIs. Differential expression of significantly expressed genes between treatments in each cell type was identified. Volcano plots compared and visualized differentially expressed genes that were upregulated and downregulated between two groups of samples. GSEA indicated that differentially expressed genes between treatments were enriched in specific pathways in each cell type (Supplement Tables S1–S9). We observed significant differences in gene expressions in GG and MG groups compared to the SG group in CD31-positive, SMA-positive cells and NeuN-positive cells (Figure 4, Figure 5 and Figure 6). In each cell type, both upregulated and downregulated genes were identified.

## 4. Discussions

GLP-2 modulates lipid handling in the gut during both feeding and fasting cycles. During food intake, GLP-2 increases lipid absorption [1,2]. During the fasted state, exogenous GLP-2 was shown to promote the release of lipids stored in the gut [3,25]. Involvement of a gut-brain neural pathway in this effect [14] highlights the significance of central regulation [14]. However, the nature of this pathway, especially its key molecular components, remains undefined. In the present study, we show that central GLP-2R and MC4R are involved in this neural pathway. Specifically, we show that blocking central GLP-2R and MC4R attenuates the effect of peripheral GLP-2 on releasing intestinally stored lipids. This finding expands the understanding of GLP-2’s mechanism of action in regulating gut lipid handling.

In the present study, we tested two potential candidate players, namely central GLP-2R and MC4R, in mediating peripheral GLP-2’s effects on mobilizing intestinally stored lipids in the fasted state. After administering icv GLP-2R antagonist and MC4R antagonist, we assessed the dynamics of lipid output from the gut in mesenteric lymph duct cannulated rats treated with peripheral GLP-2. In the absence of GLP-2R antagonist or MC4R antagonist, ip GLP-2 increased lipid output, as seen in previous studies [3,25], thus confirming the effect of peripheral GLP-2 in releasing stored lipids from the gut. Rats treated with icv GLP-2R antagonist had blunted lipid output compared to icv saline. Similarly, in rats treated with icv MC4R antagonist, there was significant attenuation in lipid output compared to icv saline. Pharmacological inhibition of central MC4R mainly affects adipose tissue metabolism; thus, chronic icv administration of SHU9119 in mice decreases brown adipose tissue activity and lipid uptake in mice [9,17]. Short-term icv administration of SHU9119 did not affect circulating TG in fasted mice, indicating a lack of effects on liver and gut lipid output [26]. Subchronic administration of GLP-2(11-33) did not elicit significant effects on triglycerides in rats [19]. These effects, therefore, are not likely attributed to the antagonists alone but rather to their attenuation of peripheral GLP-2’s effect. Built upon our previous study, these results support that central GLP-2R and MC4R are important components of this specific pathway. Our findings support an expanded model for GLP-2 mobilization of gut lipids to include a neural network that enlists the activation of GLP-2R on vagal afferent neurons, the transmission of neural signals to the brain to activate central GLP-2R and MC4R, and subsequent vagal efferent outflow to the intestine.

Although these results support key roles of central GLP-2R and MC4R in this pathway, the effects were partial, which could be explained by several possibilities. One is the existence of pathways besides gut-brain neural communication, which is in line with our previous study where disruption of the gut-brain neural communications by vagotomy did not fully abrogate GLP-2’s effects [14]. Another possibility is the activation of hypothalamic neurons via other hormones downstream of peripheral GLP-2R activation. It is also possible that peripheral GLP-2-activated POMC neurons in the hypothalamus have multiple downstream targets besides MC4R. Indeed, activation of POMC neurons by GLP-2 led to enhanced glucose tolerance and hepatic insulin sensitivity via the P13K pathway [10]. This would explain the modest effect of blocking central MC4R on GLP-2’s action in this present study. Nonetheless, this study demonstrates diminished lipid output from the gut during the post-absorptive state with pharmacological inhibition of central GLP-2R or MC4R. A recent study in mice showed a brain-gut axis in regulating intestinal fat absorption by shortening microvilli [27]. Whether chronic inhibition of central GLP-2R or MC4R has similar effects on the small intestine structure remains to be investigated.

Using digital spatial profiling, we assessed the expression of key genes within three distinct cell types of the rat jejunum. This provided a detailed picture of the molecular changes occurring with and without the inhibition of two central receptors (GLP-2R and MC4R) on the background of peripheral GLP-2. Several considerations were included in the selection of cellular markers. GLP-2 is not believed to affect lipoprotein secretion at the enterocyte level; instead, the target locations/cell types likely involve the lamina propria (where myofibroblasts are abundant) and the mesenteric lymphatics (where the lymphatic endothelium function is conferred by contractility of circular and longitudinal muscles) [13]. These regions are also richly innervated by enteric neurons, some of which regulate lipid absorption [12]. We therefore elected to use specific markers to identify the muscles (SMA), endothelium (CD31), and neurons (NeuN). Gene expression in these cell populations was differentially affected by treatments, as shown by heatmaps and volcano plots for cells positive for each marker. In CD31-positive cells, icv GLP-2R blockade led to downregulation of several genes, including *KPTN*, *KAT5*, *P4ha1*, and *Svep1,* and upregulation of several genes, including *TLL1* and *Adamts7*. These genes have important implications in atherosclerosis and lipid metabolism. *KPTN* knockdown in a human hepatocyte cell line decreased cholesterol and apoB100 secretion [28]. *KAT5* overexpression attenuated cardiomyocyte injury in rat cardiomyocytes (H9c2 cells) [29]. Overexpression of *P4ha1* increased atherosclerotic plaques in ApoE-deficient mice [30,31]. *Svep1* increased proliferation and inflammation in murine vascular smooth muscle cells and promoted atherosclerosis [32]. *TLL1* is involved in the peroxisome proliferator-activated receptor pathway, and a *TLL1* variant is related to vascular inflammation and calcification [33]. Since vascular calcification is a prominent feature of diabetic atherosclerosis [34], *TLL1* may be a strong contributor to the pathogenesis of diabetic atherosclerosis. Knockout of *Adamts7* reduced atherosclerosis in mice [35]. In SMA-positive cells, *Insm2* and *FGF7* were the important downregulated genes following icv GLP-2R blockade. Insm2 was shown to regulate insulin secretion in mice [36] and lipid metabolism in neuroblastoma cell lines [37]. *FGF7* overexpression alleviated myocardial infarction in mice [38]. *Prdx5* overexpression reduced the expression of fatty acid, suggesting that Prdx5 can regulate fatty acid oxidation in mice [39]. In SMA-positive cells, following icv GLP-2R blockade, important upregulated genes were *SFRP4* and *SLC7A7*. SFRP4 alleviates atherosclerosis in ApoE^−/−^ mice by reducing inflammation and oxidative stress and acting via the Wnt/β-catenin signaling pathway [40]. SLC7A7 is a key intermediary factor in the ATF3-SLC7A7 axis that regulates mTORC1 signaling to attenuate lipogenesis in hepatocellular carcinoma [41]. In NeuN cells, *GPR22* and *Casp1* were among the downregulated genes. Cardiomyocyte-specific overexpression of GPR22 in mice resulted in acute myocardial infarction [42]. Casp1 gene expression is increased in atherosclerotic samples compared to normal samples, as identified from the atherosclerotic dataset [43]. Upregulated genes include Cyp4a32 and SCRG1. Cyp4a32 was one of the affected genes from the PPAR signaling pathway in diabetic mice [44]. SCRG1 is identified as one of the critical diagnostic markers involved in infiltration of immune cells in atherosclerosis [45]. These affected genes also might be part of the GLP-2/GLP-2R signaling pathway in handling gut lipids; however, none of them have been studied in the intestine, especially in the context of lipid handling.

Quantitative comparison among cell types is not possible, but gene expression in CD31-specific endothelial cells and SMA-specific smooth muscle cells seems to be affected to a greater magnitude than in NeuN-positive cells. The biological significance of this is unknown, but it may suggest a mechanism whereby central GLP-2R and MC4R regulate this particular pathway mainly through modulating endothelial functions. Indeed, treatments affected key pathways relevant to endothelial functions. Particularly in endothelial cells, the vascular endothelial growth factor (VEGF)A-VEGFR2 pathway was downregulated with central GLP-2R inhibition compared with the other treatments. Intestinal lipid output is dependent on lymphatic functions [46,47], and the VEGF family is involved in regulating intestinal lipid handling [48,49]. VEGFA is involved in lymphatic pumping [50], and the VEGFA-VEGFR2 signaling pathway regulates lymphatic endothelial cell junction for intestinal lipid absorption [51]. Such a mechanism has not been demonstrated for post-absorptive lipid mobilization. Our study showed that, in rats in the presence of peripheral GLP-2, the VEGFA-VEGFR2 pathway is downregulated by central GLP-2R inhibition in the post-absorptive state. These results point to a neural pathway for peripheral GLP-2 to mobilize intestinal lipid stores during the post-absorptive state through central GLP-2R and subsequent modulation of intestinal lymphatic functions.

This study has several limitations, since observations were obtained with a small sample size, from only male animals, and under experimental settings. Future studies are needed to investigate the relative contribution of GLP-2R and MC4R in specific nuclei in the brain, the validation and functions (especially in the context of lipid handling) of responsive genes in the intestine in specific cell types, and their roles in disease conditions.

## 5. Conclusions

In conclusion, we show that brain GLP-2R and MC4R are important components of a neural pathway for peripheral GLP-2 to release lipid storage from the gut during the post-absorptive state. Dysregulated lipid handling in the gut leads to dyslipidemia and cardiovascular disease; such knowledge, therefore, may help develop therapeutic strategies by targeting such mediators for the treatment and prevention of lipid disorders and cardiovascular disease.

## Data Availability

The datasets generated and analyzed during the current study are available from the corresponding author on reasonable request. The transcriptomics datasets have been deposited in NCBI’s Gene Expression Omnibus (GEO) and are accessible through GEO Series accession number GSE286277.

## Supplementary Materials

The following supporting information can be downloaded at: https://www.mdpi.com/article/10.3390/nu17091416/s1, Figure S1: TG concentration in lymph over time following GLP-2R or MC4R blockade; Figure S2: Data quality and assay performance for spatial transcriptomics experiment; Figure S3: Principal component analysis (PCA) of variation of groups of genes among three treatments in three different cell types; Table S1: Top-ranked up (positive NES) and down (negative NES)–regulated pathways in CD31-positive cells: GG vs. SG; Table S2: Top-ranked up (positive NES) and down (negative NES)–regulated pathways in CD31-positive cells: MG vs. SG; Table S3: Top-ranked up (positive NES) and down (negative NES)–regulated pathways in CD31-positive cells: MG vs. GG; Table S4: Top-ranked up (positive NES) and down (negative NES)–regulated pathways in SMA-positive cells: GG vs. SG; Table S5: Top-ranked up (positive NES) and down (negative NES)–regulated pathways in SMA-positive cells: MG vs. SG; Table S6: Top-ranked up (positive NES) and down (negative NES)–regulated pathways in SMA-positive cells: MG vs. GG; Table S7: Top-ranked up (positive NES) and down (negative NES)–regulated pathways in NeuN-positive cells: GG vs. SG; Table S8: Top-ranked up (positive NES) and down (negative NES)–regulated pathways in NeuN-positive cells: MG vs. SG; Table S9: Top-ranked up (positive NES) and down (negative NES)–regulated pathways in NeuN-positive cells: MG vs. GG.
