# Supplementary material for: Glucagon-like Peptide-2 Acts Partially Through Central GLP-2R and MC4R in Mobilizing Stored Lipids from the Intestine
Source: Nutrients. 2025 Apr 23;17(9):1416. doi: 10.3390/nu17091416 (PMC12073721; doi:10.3390/nu17091416)
Supplement: Supplementary file 1 [file nutrients-17-01416-s001.zip › nutrients-3560714-supplementary.pdf]

Mukherjee et al.

Glucagon-like Peptide-2 Acts Partially through Central GLP-2R and MC4R in Mobilizing Stored Lipids from the Intestine

Supplement Figures and Legends

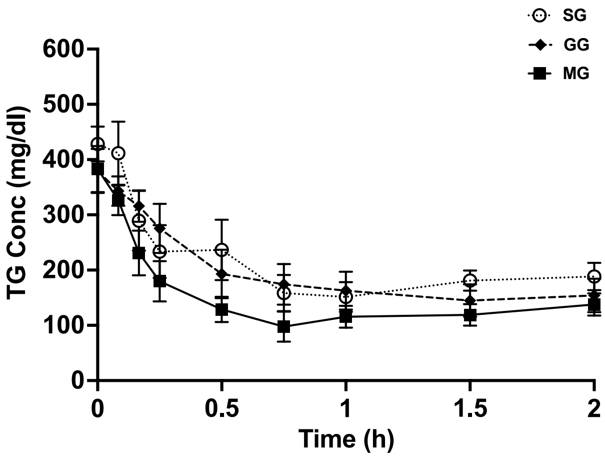

Supplement Figure S1: TG concentration in lymph over time following GLP-2R or MC4R blockade. TG concentration in lymph was measured over 2 hours. Results are expressed as mean ± SEM: icv saline + ip GLP-2 (SG), n=6; icv GLP-2(11-33) + ip GLP-2 (GG), n=9; icv SHU9119 + ip GLP-2 (MG), n=7. Two-way Repeated Measures ANOVA followed by Tukey's multiple comparison was used for statistical analysis.

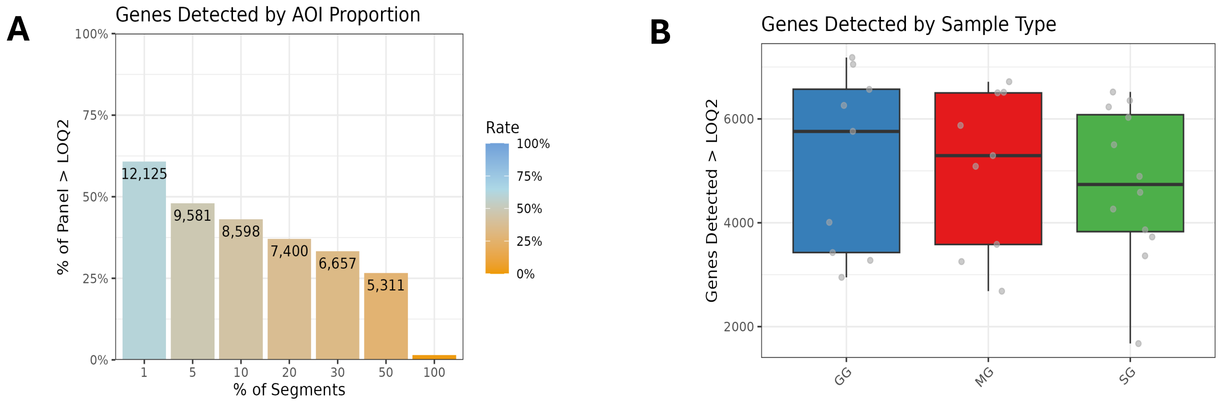

Supplement Figure S2: Data quality and assay performance for spatial transcriptomics experiment. **A.** The number of genes in different percentages of tissues*expressed* above LOQ2 (Limit of Quantification), which is the Geometric Mean of the Negative probes, multiplied by the Geometric Standard Deviation to the second power (^2). **B.** Genes were sorted by sample type in the y-axis and sorted by signal-to-noise ratio (SNR) calculated as counts/LOQ on the x-axis. SG, icv saline + ip GLP-2; GG, icv GLP-2(11-33) + ip GLP-2; MG, icv SHU9119 + ip GLP-2.

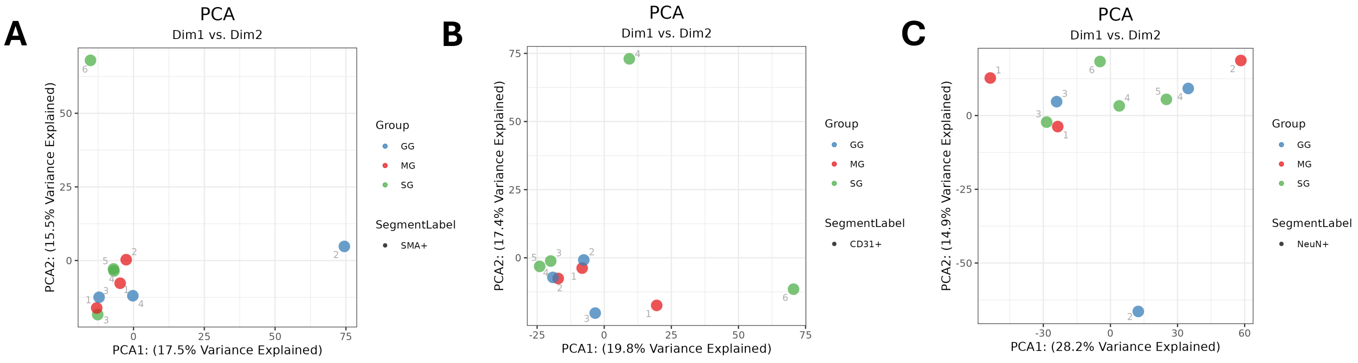

Supplement Figure S3: Principal component analysis (PCA) of variation of groups of genes among three treatments in three different cell types: The plot of the first two principal components (PCA1 and PCA2), calculated from the expression data of the genes with the highest variance across all samples. Axis labels indicate the percentage of total variance explained by each component. GG group (icv GLP-2(11-33) + ip GLP-2) was denoted by blue; MG group (icv SHU9119 + ip GLP-2) group was denoted by red; SG group (icv saline + ip GLP-2 saline) was denoted by green symbols. A. SMA+ cell type; B. CD31+ cell type; C. NeuN+ cell type.

Supplementary Table 1. Top-ranked up (positive NES) and down (negative NES) – regulated pathways in CD31-positive cells: GG vs SG

Pathway	NES	P value	P adj
RAB GEFs exchange GTP for GDP on RABs	1.4371	4.1E-02	8.7E-01
Rab regulation of trafficking	1.3516	5.3E-02	8.7E-01
Amplification of signal from unattached kinetochores via a MAD2 inhibitory signal	1.3217	8.0E-02	8.7E-01
Amplification of signal from the kinetochores	1.3217	8.0E-02	8.7E-01
Mitochondrial translation elongation	1.2979	1.0E-01	8.7E-01
Resolution of Sister Chromatid Cohesion	1.2888	9.2E-02	8.7E-01
The citric acid (TCA) cycle and respiratory electron transport	1.2716	9.9E-02	8.7E-01
Metabolism of water-soluble vitamins and cofactors	1.2684	1.0E-01	8.7E-01
Mitotic Prophase	1.2613	1.1E-01	8.7E-01
Mitochondrial translation termination	1.2526	1.3E-01	8.7E-01
Mitochondrial translation	1.2311	1.5E-01	8.7E-01
Protein-protein interactions at synapses	1.1997	1.9E-01	8.7E-01
RAF/MAP kinase cascade	-1.2558	4.3E-02	8.7E-01
MHC class II antigen presentation	-1.3060	6.7E-02	8.7E-01
COPI-mediated anterograde transport	-1.3122	7.6E-02	8.7E-01
Transmission across Chemical Synapses	-1.3866	2.4E-02	7.9E-01
Opioid Signalling	-1.4606	5.4E-02	8.7E-01
Factors involved in megakaryocyte development and platelet production	-1.4768	1.8E-02	7.0E-01
Signaling by NTRKs	-1.4838	3.8E-02	8.7E-01
Neurotransmitter receptors and postsynaptic signal transmission	-1.5393	8.8E-03	4.2E-01
Signaling by VEGF	-1.5977	4.4E-03	3.8E-01
Integration of energy metabolism	-1.5987	9.0E-03	4.2E-01
Fcgamma receptor (FCGR) dependent phagocytosis	-1.6114	4.9E-03	3.8E-01
VEGFA-VEGFR2 Pathway	-1.7253	2.1E-03	3.8E-01

NES, normalized enrichment scores.

Supplementary Table 2. Top-ranked up (positive NES) and down (negative NES) – regulated pathways in CD31-positive cells: MG vs SG

Pathway	NES	P value	P adj
Mitotic Spindle Checkpoint	1.7114	1.4E-03	3.3E-01
Amplification of signal from unattached kinetochores via a MAD2 inhibitory signal	1.6118	7.2E-03	3.7E-01
Amplification of signal from the kinetochores	1.6118	7.2E-03	3.7E-01
EML4 and NUDC in mitotic spindle formation	1.5943	8.0E-03	3.7E-01
Separation of Sister Chromatids	1.4683	1.1E-02	4.3E-01
Resolution of Sister Chromatid Cohesion	1.4259	3.9E-02	5.8E-01
Cargo recognition for clathrin-mediated endocytosis	1.4256	4.0E-02	5.8E-01
RHO GTPases Activate Formins	1.3979	3.7E-02	5.8E-01
Cell Cycle Checkpoints	1.3937	1.6E-02	4.6E-01
Potassium Channels	1.3923	5.9E-02	6.8E-01
Signaling by TGFB family members	1.3846	4.9E-02	6.7E-01
Class I MHC mediated antigen processing & presentation	1.3772	1.8E-02	4.6E-01
HDR through Homologous Recombination (HRR) or Single Strand Annealing (SSA)	-1.1170	2.3E-01	7.6E-01
Toll Like Receptor 4 (TLR4) Cascade	-1.1238	2.3E-01	7.6E-01
DNA Double-Strand Break Repair	-1.1260	1.7E-01	7.6E-01
Developmental Biology	-1.1282	2.2E-01	7.6E-01
COPI-mediated anterograde transport	-1.1355	2.0E-01	7.6E-01
Asparagine N-linked glycosylation	-1.1450	1.3E-01	7.6E-01
Toll-like Receptor Cascades	-1.1779	2.0E-01	7.6E-01
Toll Like Receptor 9 (TLR9) Cascade	-1.1986	1.6E-01	7.6E-01
Phase I - Functionalization of compounds	-1.2581	1.1E-01	7.6E-01
Axon guidance	-1.2714	2.5E-02	5.2E-01
Nervous system development	-1.2714	2.5E-02	5.2E-01
Biosynthesis of the N-glycan precursor (dolichol lipid-linked oligosaccharide, LLO) and transfer to a nascent protein	-1.6907	3.4E-03	3.7E-01

NES, normalized enrichment scores.

Supplementary Table 3. Top-ranked up (positive NES) and down (negative NES) – regulated pathways in CD31-positive cells: MG vs GG

Pathway	NES	P value	P adj
mRNA Splicing	1.5641	3.8E-03	6.0E-01
Mitotic Spindle Checkpoint	1.5457	1.5E-02	6.0E-01
mRNA Splicing - Major Pathway	1.5412	1.1E-02	6.0E-01
Integration of energy metabolism	1.5154	2.0E-02	6.0E-01
Nucleotide Excision Repair	1.4632	3.7E-02	6.0E-01
Processing of Capped Intron-Containing Pre-mRNA	1.4569	8.7E-03	6.0E-01
VEGFA-VEGFR2 Pathway	1.4492	2.7E-02	6.0E-01
Degradation of the extracellular matrix	1.4483	3.9E-02	6.0E-01
Opioid Signalling	1.4329	5.2E-02	6.0E-01
Neurotransmitter receptors and postsynaptic signal transmission	1.4083	3.5E-02	6.0E-01
Transmission across Chemical Synapses	1.3917	3.2E-02	6.0E-01
DNA Replication Pre-Initiation	1.3714	6.7E-02	6.0E-01
Homology Directed Repair	-1.0581	3.4E-01	8.2E-01
HDR through Homologous Recombination (HRR) or Single Strand Annealing (SSA)	-1.0770	3.2E-01	8.2E-01
Developmental Biology	-1.0920	1.9E-01	7.2E-01
Stimuli-sensing channels	-1.1185	2.6E-01	7.6E-01
Axon guidance	-1.1263	1.8E-01	7.2E-01
Nervous system development	-1.1263	1.8E-01	7.2E-01
Negative regulation of the PI3K/AKT network	-1.1379	2.4E-01	7.3E-01
Toll-like Receptor Cascades	-1.1429	1.8E-01	7.2E-01
Rab regulation of trafficking	-1.1482	1.9E-01	7.2E-01
PI5P, PP2A and IER3 Regulate PI3K/AKT Signaling	-1.1781	1.7E-01	7.2E-01
DNA Double-Strand Break Repair	-1.2093	1.2E-01	6.7E-01
RAB GEFs exchange GTP for GDP on RABs	-1.3081	1.2E-01	6.7E-01

NES, normalized enrichment scores.

Supplementary Table 4. Top-ranked up (positive NES) and down (negative NES) – regulated pathways in SMA-positive cells: GG vs SG

Pathway	NES	P value	P adj
Protein localization	1.5699	7.3E-03	1.0E+00
trans-Golgi Network Vesicle Budding	1.4101	3.7E-02	1.0E+00
G2/M Checkpoints	1.3384	5.4E-02	1.0E+00
Signaling by TGF-beta Receptor Complex	1.3055	8.5E-02	1.0E+00
Downstream TCR signaling	1.2755	9.9E-02	1.0E+00
Regulation of mRNA stability by proteins that bind AU-rich elements	1.2635	1.0E-01	1.0E+00
Metabolism of nucleotides	1.2533	1.1E-01	1.0E+00
MAPK6/MAPK4 signaling	1.2412	1.3E-01	1.0E+00
ABC-family proteins mediated transport	1.1934	1.3E-01	1.0E+00
Cell Cycle Checkpoints	1.1778	1.3E-01	1.0E+00
Activation of APC/C and APC/C:Cdc20 mediated degradation of mitotic proteins	1.1765	1.8E-01	1.0E+00
APC/C:Cdh1 mediated degradation of Cdc20 and other APC/C:Cdh1 targeted proteins in late mitosis/early G1	1.1569	2.0E-01	1.0E+00
RAB GEFs exchange GTP for GDP on RABs	-1.2152	1.5E-01	1.0E+00
Programmed Cell Death	-1.2386	1.2E-01	1.0E+00
Keratinization	-1.2903	9.0E-02	1.0E+00
Potassium Channels	-1.2990	8.3E-02	1.0E+00
Regulation of Insulin-like Growth Factor (IGF) transport and uptake by Insulin-like Growth Factor Binding Proteins (IGFBPs)	-1.3051	7.0E-02	1.0E+00
Biosynthesis of the N-glycan precursor (dolichol lipid-linked oligosaccharide, LLO) and transfer to a nascent protein	-1.3075	1.1E-01	1.0E+00
Intracellular signaling by second messengers	-1.3127	4.1E-02	1.0E+00
Post-translational protein phosphorylation	-1.3310	6.2E-02	1.0E+00
Metabolism of carbohydrates	-1.3749	2.8E-02	1.0E+00
Opioid Signalling	-1.4522	3.0E-02	1.0E+00
O-linked glycosylation	-1.4712	1.8E-02	1.0E+00
Glucose metabolism	-1.5283	2.1E-02	1.0E+00

NES, normalized enrichment scores.

Supplementary Table 5. Top-ranked up (positive NES) and down (negative NES) – regulated pathways in SMA-positive cells: MG vs SG

Pathway	NES	P value	P adj
Signaling by TGF-beta Receptor Complex	1.4782	2.4E-02	7.5E-01
Global Genome Nucleotide Excision Repair (GG-NER)	1.4424	3.5E-02	7.5E-01
Nucleotide Excision Repair	1.4153	4.2E-02	7.5E-01
Protein localization	1.3584	7.2E-02	9.0E-01
Signaling by TGFB family members	1.2659	1.1E-01	9.8E-01
The citric acid (TCA) cycle and respiratory electron transport	1.2284	9.8E-02	9.8E-01
trans-Golgi Network Vesicle Budding	1.1696	2.0E-01	9.9E-01
Toll Like Receptor 9 (TLR9) Cascade	1.1568	2.1E-01	9.9E-01
Apoptosis	1.1505	2.1E-01	9.9E-01
Cargo recognition for clathrin-mediated endocytosis	1.1481	2.1E-01	9.9E-01
Mitotic Spindle Checkpoint	1.1018	2.8E-01	9.9E-01
Mitotic Prometaphase	1.1017	2.8E-01	9.9E-01
G alpha (q) signalling events	-1.2948	5.9E-02	8.2E-01
Hemostasis	-1.2977	2.5E-02	7.5E-01
G alpha (s) signalling events	-1.3112	6.0E-02	8.2E-01
Regulation of mRNA stability by proteins that bind AU-rich elements	-1.3187	9.6E-02	9.8E-01
Beta-catenin independent WNT signaling	-1.3227	5.4E-02	8.2E-01
Signaling by FGFR	-1.3239	8.7E-02	9.8E-01
Class A/1 (Rhodopsin-like receptors)	-1.3337	2.3E-02	7.5E-01
Muscle contraction	-1.3558	3.9E-02	7.5E-01
Peptide ligand-binding receptors	-1.4076	3.6E-02	7.5E-01
PTEN Regulation	-1.4207	4.1E-02	7.5E-01
Platelet homeostasis	-1.5422	1.7E-02	7.5E-01
Potassium Channels	-1.6098	5.4E-03	7.5E-01

NES, normalized enrichment scores.

Supplementary Table 6. Top-ranked up (positive NES) and down (negative NES) – regulated pathways in SMA-positive cells: MG vs GG

Pathway	NES	P value	P adj
Apoptosis	1.5981	1.1E-02	4.3E-01
Metabolism of carbohydrates	1.4485	9.3E-03	4.3E-01
Programmed Cell Death	1.3870	4.8E-02	7.3E-01
PI5P, PP2A and IER3 Regulate PI3K/AKT Signaling	1.3829	6.2E-02	7.3E-01
RAB GEFs exchange GTP for GDP on RABs	1.3781	6.4E-02	7.3E-01
Global Genome Nucleotide Excision Repair (GG-NER)	1.3088	8.1E-02	7.3E-01
Negative regulation of the PI3K/AKT network	1.2916	9.1E-02	7.5E-01
Glucose metabolism	1.2452	1.5E-01	7.6E-01
Biosynthesis of the N-glycan precursor (dolichol lipid-linked oligosaccharide, LLO) and transfer to a nascent protein	1.2265	1.5E-01	7.6E-01
Opioid Signalling	1.1759	2.0E-01	7.6E-01
Signaling by Nuclear Receptors	1.1726	1.5E-01	7.6E-01
Phase I - Functionalization of compounds	1.1692	2.0E-01	7.6E-01
Assembly of the pre-replicative complex	-1.3923	6.1E-02	7.3E-01
G2/M Checkpoints	-1.4104	2.7E-02	6.4E-01
Muscle contraction	-1.4126	2.6E-02	6.4E-01
Transcriptional regulation by RUNX1	-1.4188	1.9E-02	6.3E-01
APC/C-mediated degradation of cell cycle proteins	-1.4268	5.4E-02	7.3E-01
Regulation of mitotic cell cycle	-1.4268	5.4E-02	7.3E-01
Activation of APC/C and APC/C:Cdc20 mediated degradation of mitotic proteins	-1.4496	3.4E-02	7.0E-01
TNFR2 non-canonical NF-kB pathway	-1.4844	3.6E-02	7.0E-01
Platelet homeostasis	-1.4950	2.8E-02	6.4E-01
APC/C:Cdh1 mediated degradation of Cdc20 and other APC/C:Cdh1 targeted proteins in late mitosis/early G1	-1.5909	1.1E-02	4.3E-01
MAPK6/MAPK4 signaling	-1.6488	7.5E-03	4.3E-01
Regulation of mRNA stability by proteins that bind AU-rich elements	-1.6643	5.5E-03	4.3E-01

NES, normalized enrichment scores.

Supplementary Table 7. Top-ranked up (positive NES) and down (negative NES) – regulated pathways in NeuN-positive cells: GG vs SG

Pathway	NES	P value	P adj
Nonsense Mediated Decay (NMD) enhanced by the Exon Junction Complex (EJC)	1.8874	2.4E-04	1.8E-02
Nonsense-Mediated Decay (NMD)	1.8874	2.4E-04	1.8E-02
The citric acid (TCA) cycle and respiratory electron transport	1.8269	1.3E-04	1.8E-02
Respiratory electron transport	1.8174	1.1E-03	4.4E-02
Respiratory electron transport, ATP synthesis by chemiosmotic coupling, and heat production by uncoupling proteins.	1.8163	6.3E-04	3.6E-02
Nonsense Mediated Decay (NMD) independent of the Exon Junction Complex (EJC)	1.7679	1.5E-03	4.5E-02
L13a-mediated translational silencing of Ceruloplasmin expression	1.7417	8.5E-04	3.9E-02
trans-Golgi Network Vesicle Budding	1.7276	2.9E-03	6.7E-02
SRP-dependent cotranslational protein targeting to membrane	1.7036	3.0E-03	6.7E-02
GTP hydrolysis and joining of the 60S ribosomal subunit	1.6984	1.6E-03	4.5E-02
Formation of a pool of free 40S subunits	1.6531	5.2E-03	8.0E-02
Cap-dependent Translation Initiation	1.6003	8.6E-03	1.1E-01
Class A/1 (Rhodopsin-like receptors)	-1.4132	1.5E-02	1.7E-01
Cellular response to hypoxia	-1.4347	4.9E-02	3.6E-01
Hedgehog 'on' state	-1.4504	5.1E-02	3.7E-01
RAF/MAP kinase cascade	-1.4655	3.6E-03	6.7E-02
MAPK family signaling cascades	-1.4661	3.8E-03	6.7E-02
Potassium Channels	-1.4696	2.7E-02	2.5E-01
MAPK1/MAPK3 signaling	-1.4738	3.4E-03	6.7E-02
Degradation of the extracellular matrix	-1.5233	1.8E-02	1.8E-01
Transcriptional regulation by RUNX3	-1.5284	2.2E-02	2.1E-01
Interleukin-1 signaling	-1.5392	1.1E-02	1.3E-01
KEAP1-NFE2L2 pathway	-1.6061	1.2E-02	1.4E-01
PTEN Regulation	-1.6161	4.9E-03	8.0E-02

NES, normalized enrichment scores.

Supplementary Table 8. Top-ranked up (positive NES) and down (negative NES) – regulated pathways in NeuN-positive cells: MG vs SG

Pathway	NES	P value	P adj
Glycerophospholipid biosynthesis	1.3727	5.9E-02	5.7E-01
SUMOylation of DNA damage response and repair proteins	1.3582	7.6E-02	6.2E-01
SUMO E3 ligases SUMOylate target proteins	1.3288	6.8E-02	6.2E-01
Amplification of signal from unattached kinetochores via a MAD2 inhibitory signal	1.3116	1.0E-01	6.7E-01
Amplification of signal from the kinetochores	1.3116	1.0E-01	6.7E-01
SUMOylation	1.3089	8.1E-02	6.2E-01
Biosynthesis of the N-glycan precursor (dolichol lipid-linked oligosaccharide, LLO) and transfer to a nascent protein	1.3020	1.2E-01	7.3E-01
Resolution of Sister Chromatid Cohesion	1.2767	1.1E-01	7.1E-01
Cilium Assembly	1.2371	1.2E-01	7.4E-01
Metabolism of lipids	1.2310	5.6E-02	5.6E-01
Phospholipid metabolism	1.2243	1.4E-01	8.0E-01
RHO GTPase Effectors	1.2098	1.3E-01	7.5E-01
Interleukin-1 family signaling	-1.3306	9.4E-02	6.7E-01
MAPK1/MAPK3 signaling	-1.3323	1.4E-02	4.4E-01
Class A/1 (Rhodopsin-like receptors)	-1.3611	8.7E-03	4.4E-01
Clathrin-mediated endocytosis	-1.3940	3.9E-02	4.4E-01
Intracellular signaling by second messengers	-1.4106	9.7E-03	4.4E-01
Interleukin-1 signaling	-1.4337	2.8E-02	4.4E-01
PIP3 activates AKT signaling	-1.4672	6.0E-03	4.4E-01
Death Receptor Signalling	-1.4829	1.2E-02	4.4E-01
Potassium Channels	-1.5139	2.1E-02	4.4E-01
PI5P, PP2A and IER3 Regulate PI3K/AKT Signaling	-1.5436	1.7E-02	4.4E-01
Negative regulation of the PI3K/AKT network	-1.5464	9.9E-03	4.4E-01
Peptide ligand-binding receptors	-1.5571	2.3E-03	4.4E-01

NES, normalized enrichment scores.

Supplementary Table 9. Top-ranked up (positive NES) and down (negative NES) – regulated pathways in NeuN-positive cells: MG vs GG

Pathway	NES	P value	P adj
Biosynthesis of the N-glycan precursor (dolichol lipid-linked oligosaccharide, LLO) and transfer to a nascent protein	1.4406	3.7E-02	3.4E-01
TNFR2 non-canonical NF-kB pathway	1.4264	4.2E-02	3.4E-01
Sensory Perception	1.3088	6.0E-02	4.4E-01
Degradation of the extracellular matrix	1.2305	1.5E-01	7.9E-01
SLC-mediated transmembrane transport	1.2179	1.3E-01	7.4E-01
Metabolism of vitamins and cofactors	1.2178	1.5E-01	7.9E-01
Transport of small molecules	1.1923	7.4E-02	4.7E-01
Ion channel transport	1.1859	1.7E-01	8.6E-01
Regulation of mRNA stability by proteins that bind AU-rich elements	1.1288	2.5E-01	1.0E+00
Cilium Assembly	1.1268	2.5E-01	1.0E+00
S Phase	1.1120	2.8E-01	1.0E+00
Metabolism of nucleotides	1.1034	3.0E-01	1.0E+00
Signaling by NTRKs	-1.6159	9.9E-03	1.1E-01
Negative regulation of the PI3K/AKT network	-1.6309	5.6E-03	7.2E-02
SRP-dependent cotranslational protein targeting to membrane	-1.7455	1.6E-03	3.1E-02
GTP hydrolysis and joining of the 60S ribosomal subunit	-1.7481	6.4E-04	1.6E-02
Formation of a pool of free 40S subunits	-1.7506	4.8E-04	1.6E-02
Nonsense Mediated Decay (NMD) enhanced by the Exon Junction Complex (EJC)	-1.7752	4.0E-04	1.6E-02
Nonsense-Mediated Decay (NMD)	-1.7752	4.0E-04	1.6E-02
L13a-mediated translational silencing of Ceruloplasmin expression	-1.8131	2.5E-04	1.6E-02
Nonsense Mediated Decay (NMD) independent of the Exon Junction Complex (EJC)	-1.8238	5.7E-04	1.6E-02
Respiratory electron transport, ATP synthesis by chemiosmotic coupling, and heat production by uncoupling proteins.	-1.8443	4.7E-04	1.6E-02
The citric acid (TCA) cycle and respiratory electron transport	-1.8568	9.2E-05	1.6E-02
trans-Golgi Network Vesicle Budding	-1.8713	3.9E-04	1.6E-02

NES, normalized enrichment scores.
